# Structural analysis of nanocrystals by pair distribution function combining electron diffraction with crystal tilting

**DOI:** 10.1107/S2052252524001064

**Published:** 2024-02-16

**Authors:** Linshuo Guo, Shitao Wu, Zhengyang Zhou, Yanhang Ma

**Affiliations:** a School of Physical Science and Technology, and Shanghai Key Laboratory of High-resolution Electron Microscopy, ShanghaiTech University, Shanghai 201210, People’s Republic of China; b State Key Laboratory of High Performance Ceramics and Superfine Microstructure, Shanghai Institute of Ceramics, Chinese Academy of Sciences, Shanghai 200050, People’s Republic of China; Istituto Italiano di Tecnologia, Italy

**Keywords:** electron pair distribution functions, local structures, crystal tilting, nanoparticles, PDF refinement

## Abstract

As an important characterization method, pair distribution function (PDF) has been extensively used in structural analysis of nanomaterials, providing key insights into the degree of crystallinity, atomic structure, local disorder *etc*. The collection of scattering signals with good statistics is necessary for a reliable structural analysis. However, current conventional electron diffraction experiments using PDF (ePDF) are limited in their ability to acquire continuous diffraction rings for large nanoparticles. Herein, a new method – tilt-ePDF – is proposed to improve the data quality and compatibility of ePDF by a combination of electron diffraction and specimen tilting. In the present work, a tilt-series of electron diffraction patterns was collected from gold nanoparticles with three different sizes and a standard sample polycrystalline aluminium film for ePDF analysis. The results show that tilt-ePDF can not only enhance the continuity of diffraction rings, but can also improve the signal-to-noise ratio in the high scattering angle range. As a result, compared with conventional ePDF data, tilt-ePDF data provide structure parameters with a better accuracy and lower residual factors in the refinement against the crystal structure. This method provides a new way of utilizing ePDF to obtain accurate local structure information from nanoparticles.

## Abstract 2

A new method is proposed to enhance the continuity of diffraction rings and improve the signal-to-noise ratio in electron diffraction data for electron pair distribution function (ePDF) analysis.

## 1. Introduction

The precise determination of the atomic structure of nanomaterials is one of the most important underlying scientific challenges in materials science (Billinge & Levin, 2007 ▸; Egami & Billinge, 2012 ▸; Jadzinsky *et al.*, 2007 ▸); it is significant in the elucidation of structure–property correlations, the exploration of formation mechanisms and the guidance of new functional material synthesis. For some well crystallized nanocrystals, structural information can be obtained by single-crystal/powder X-ray diffraction (Hauptman, 1986 ▸; David & Shankland, 2008 ▸), 3D electron diffraction (Zhang *et al.*, 2010 ▸; Gemmi *et al.*, 2019 ▸) or high-resolution electron microscopic imaging (Zhang *et al.*, 2018 ▸). For some amorphous materials, polycrystalline materials and extremely tiny nanoparticles, the above-mentioned methods exhibit their respective limitations in revealing accurate structure parameters. Pair distribution function (PDF) (Egami & Billinge, 2012 ▸; Warren, 1990 ▸; Billinge, 2008 ▸; Masadeh *et al.*, 2007 ▸) serves as a robust method in the realm of crystallography, facilitating a rather powerful elucidation of the atomic structures of these materials.

Over the years of its development, PDF has mostly been applied to organic crystals (Castillo-Blas *et al.*, 2020 ▸; Prill *et al.*, 2016 ▸), nanomaterials (Kodama *et al.*, 2006 ▸; Willinger *et al.*, 2017 ▸) and molecular materials (Terban *et al.*, 2016 ▸; Terban & Billinge, 2022 ▸). PDF analysis is typically conducted with three sources: high-energy X-rays (Billinge & Kanatzidisb, 2004 ▸; Tyrsted *et al.*, 2012 ▸; Grangeon *et al.*, 2017 ▸; Anker *et al.*, 2021 ▸), neutrons (Frandsen *et al.*, 2014 ▸) and electrons (Gorelik, 2018 ▸; Gorelik *et al.*, 2019 ▸; Corrêa *et al.*, 2021 ▸; Souza *et al.*, 2021 ▸; Schmidt *et al.*, 2023 ▸). Compared with high-energy X-rays and neutrons, electrons are easy to access using a transmission electron microscope (TEM) available in many laboratories. Because of the strong interaction between electrons and matter, nanosized samples are sufficient for electron pair distribution function (ePDF) (Cowley, 1992 ▸; Abeykoon *et al.*, 2012 ▸; Mu *et al.*, 2013 ▸). High spatial resolution can be also achieved using nanobeam electron diffraction (Mu *et al.*, 2019 ▸). Meanwhile, collecting data by TEM will require less sample, which is beneficial in cases where samples are difficult to synthesize in large quantities. Furthermore, TEM has the capability to observe specimens in real space, including the morphology and size of nanoparticles, as well as atomic resolution images. It provides key information in real space that cannot be obtained from X-rays or neutrons.

A basic ePDF characterization process contains three steps including electron diffraction data collection, data processing and structure refinement. Data collection is usually carried out by capturing selected-area electron diffraction (SAED) patterns consisting of diffraction rings, followed by further data processing (Tran *et al.*, 2017 ▸; Shanmugam *et al.*, 2017 ▸; Shi *et al.*, 2019 ▸). The resultant PDF curves can be refined against structural models using specific refining algorithms (Anker *et al.*, 2021 ▸; Farrow *et al.*, 2014 ▸; Li *et al.*, 2017 ▸). Parameters including the completeness and resolution of the collected data will directly affect the quality of the PDF results. Hence, in order to procure accurate PDF data with high resolution, it is imperative to collect scattering data with a good signal-to-noise ratio up to a large *Q* value (scattering vector). However, because of the fast damping of the electron atomic scattering factor, the intensity of the diffraction rings become much weaker at high *Q* values. The diffraction data of some materials may not be optimal, especially for large nanoparticles (>10 nm) (Abeykoon *et al.*, 2012 ▸; Junior *et al.*, 2021 ▸), resulting in PDF curves with low quality. Therefore, different ePDF methods based on electron diffraction (ED) (Abeykoon *et al.*, 2012 ▸; Schleder *et al.*, 2019 ▸; Das *et al.*, 2017 ▸; Hoque *et al.*, 2019 ▸) have been proposed for data quality improvement which, to a greater extent, have promoted the capability and applicability of ePDF to a wide range of materials.

Despite the extraordinary efforts devoted to the ePDF method development, the hurdles of improving data quality and specimen size constraints persist, impeding its further applications. Conventionally, the sizes of specimen particles (Kodama *et al.*, 2006 ▸; Tran *et al.*, 2016 ▸) suitable for ePDF analysis are limited (usually less than 10 nm). On the one hand, a large particle thickness might bring strong multiple scattering effects, which affects the acquisition of a reliable scattering intensity profile (Vincent & Midgley, 1994 ▸). On the other hand, in each data acquisition experiment, the sampling area is physically predetermined by selected-area apertures. For ultra-small nanoparticles, the deviation from ideal data may be negligible, whereas for large nanoparticles, discontinuous diffraction rings with dramatic intensity variation would be obtained, leading to a prominent reduction in both completeness and quality of data.

Herein, to increase the continuity of diffraction rings of nanoparticles with large particle sizes and obtain high-quality ePDF data, we propose a new approach that involves the collection of electron diffraction rings via crystal tilting, termed tilt-ePDF. A tilt-series of ED patterns was collected and overlapped to obtain diffraction data for gold nanoparticles (AuNPs) of different sizes and polycrystalline aluminium film. Compared with conventional ED and precession ED (PED), the diffraction data obtained through tilting showed a significant improvement in the signal-to-noise ratio and enhanced continuity of diffraction rings. The refinement of these samples was then conducted against standard structural models, which showed that the tilt-ePDF data can decrease the residual factors of refinement, suggesting the promising application of tilt-ePDF.

## 2. Materials and methods

### 2.1. AUNP and aluminium film preparation

#### 2.1.1. Synthesis of AuNPs of different sizes

To obtain AuNPs of different sizes, the Turkevich–Frens reaction system (Bastús *et al.*, 2011 ▸; Zhao *et al.*, 2013 ▸) was adopted to synthesize spherical AuNPs of progressive sizes. Raw materials for AuNPs synthesis, tris­odium citrate (Na_3_Cit, 99%, Adamas Co. Ltd) and gold (III) chloride trihydrate (HAuCl_4_·3H_2_O, 99.9%+, Adamas Co. Ltd) were firstly dissolved in deionized water to prepare 2.2 and 25 m*M* solutions, respectively. As a typical protocol, 150 ml of 2.2 m*M* Na_3_Cit solution was first added to a 500 ml three-necked round-bottomed flask and then heated in an oil bath with stirring using a magnetic mixer to ensure temperature stability. After reaching 373 K, 1 ml of HAuCl_4_·3H_2_O (25 m*M*) was added to the flask. The color of the solution changed from yellow to pink after about 10 min. After, 3 ml solution was taken by a pipette and transferred into a 10 ml centrifugal tube to cool down naturally, denoted as the first-generation gold nanoparticles (first AuNPs).

The second-generation gold nanoparticles (second AuNPs) were synthesized based on the first AuNPs. The remaining solution in the three-neck flask was cooled to 363 K before adding 1 ml of HAuCl_4_·3H_2_O (25 m*M*). After 30 min, 2 ml of HAuCl_4_·3H_2_O (25 m*M*) was added to the solution. When the reaction in the solution had proceeded for 30 min at 363 K, 3 ml of the solution was removed for cooling, and the second AuNPs were obtained. To synthesize third-generation gold nanoparticles (third AuNPs), 55 ml was removed from the second solution from the flask and 53 ml ultrapure water and 2 ml of Na_3_Cit (2.2 m*M*) was added. 1 ml of HAuCl_4_·3H_2_O (25 m*M*) was added when the solution was stabilized at 363 K. The subsequent operation was the same as the synthesis of the second AuNPs: the solution reaction took 30 min and then 2 ml HAuCl_4_·3H_2_O (25 m*M*) was added. After waiting for 30 min, 3 ml of the solution was removed to cool down in air, and the third AuNPs were obtained.

#### 2.1.2. Evaporated aluminium film

Polycrystalline aluminium (Al) film was purchased from Ted Pella Inc (Table S1 of the supporting information) and distributed on a 3 mm TEM grid. The specimen was used without further purification. In addition, three samples of Al films with different thicknesses were prepared. TEM grids coated with ultra-thin carbon films were placed into a magnetron sputtering instrument and the sputter times were set to 121, 363 and 909 s to produce Al films on the TEM grids; as a result, three Al film samples with thicknesses of ∼20, 60 and 150 nm, respectively, were obtained.

### 2.2. Sample preparation

The three AuNPs samples (first, second and third AuNPs) of different sizes were all colloidal suspensions. However, because of their high concentrations, the particles distributed in the solution agglomerated easily. Therefore, the suspensions were pretreated in an ultrasonic cleaner for 30 min to preserve the suspension and avoid agglomeration of nanoparticles on the carbon films. Then, 2 µl of the suspension was dropped onto a copper grid loaded with ultra-thin carbon film (200 mesh), and the copper grids were used in TEM characterization after waiting 24 h for them to dry in air.

### 2.3. Data collection of single-ePDF, PED-ePDF and tilt-ePDF

The copper grids were loaded on a high-tilt specimen holder, which enables a large tilting angle range (±70°). Since the gold and aluminium specimens are rather stable under electron beams, a high electron dose (Table S2) was applied to collect diffraction patterns to ensure a high resolution and good signal-to-noise ratio. A beam stopper was used to block the central spot to avoid damage to the camera, and the exposure time was set to 500 ms. The diffraction patterns were recorded using 32-bit or 16-bit images to reach a high dynamic range (Table S3). All original data, including TEM images and diffraction patterns, were obtained using a Rio-16 detector equipped on a Jeol JEM-F200 TEM and a TVIPS (XF416) camera on a Jeol JEM-2100Plus at room temperature. The accelerating voltage was 200 kV (λ = 0.0251 Å).

**Figure 1:**
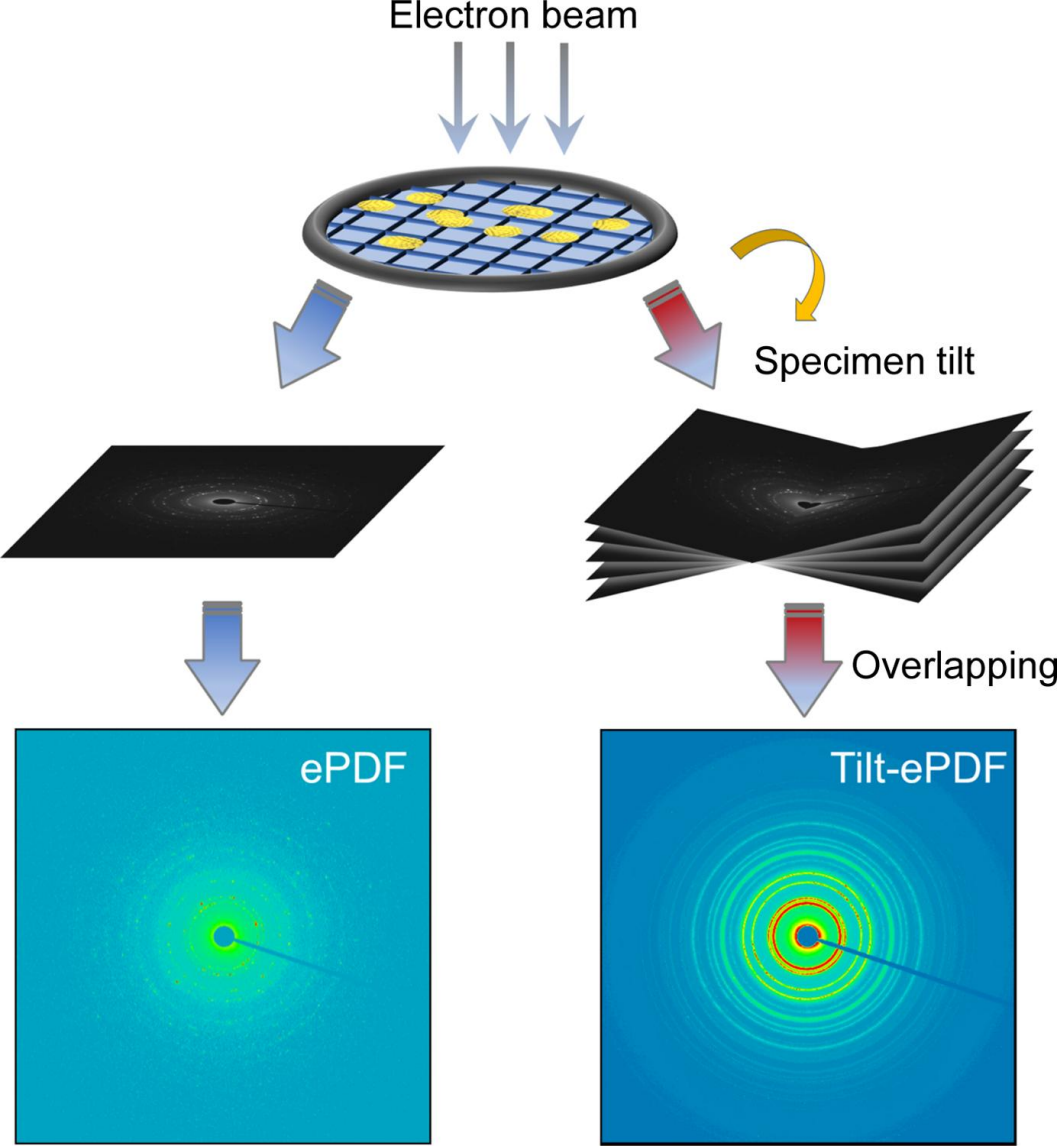
Schematic of different data collection methods of ePDF.

In the present work, diffraction data were acquired by either using a single ED frame without or with PED (single-ePDF, PED-ePDF) or merging a tilt-series of ED frames (tilt-ePDF). A schematic of the data collected is shown in Fig. 1 ▸. Two different TEM instruments were used in the experiments, JEOL JEM-F200 and JEOL JEM-2100Plus, where the largest SAED apertures were around 1.17 and 3.26 µm, respectively (Fig. S1 of the supporting information). A camera length of 250 mm was used to cover a wide range of *Q*-values up to 16.50 Å^−1^. For tilt-ePDF, a tilt-series of ED patterns was acquired from almost the same region but at different angles using a high-tilt holder. Of note, the change of selected areas is unavoidable herein while the shift of specimen can be minimized by careful alignment. The tilting angle ranges and speed for each dataset of tilt-ePDF were listed in Table S2. *DigitalMicrograph* suite (*DM*, Digital Micrograph Gatan, Pleasanton, California, USA) plugin ‘*Image Alignment*’ was then used to correct the beam center of each image by a bandpass algorithm.

## 3. Results

### 3.1. TEM images and electron diffraction

**Figure 2:**
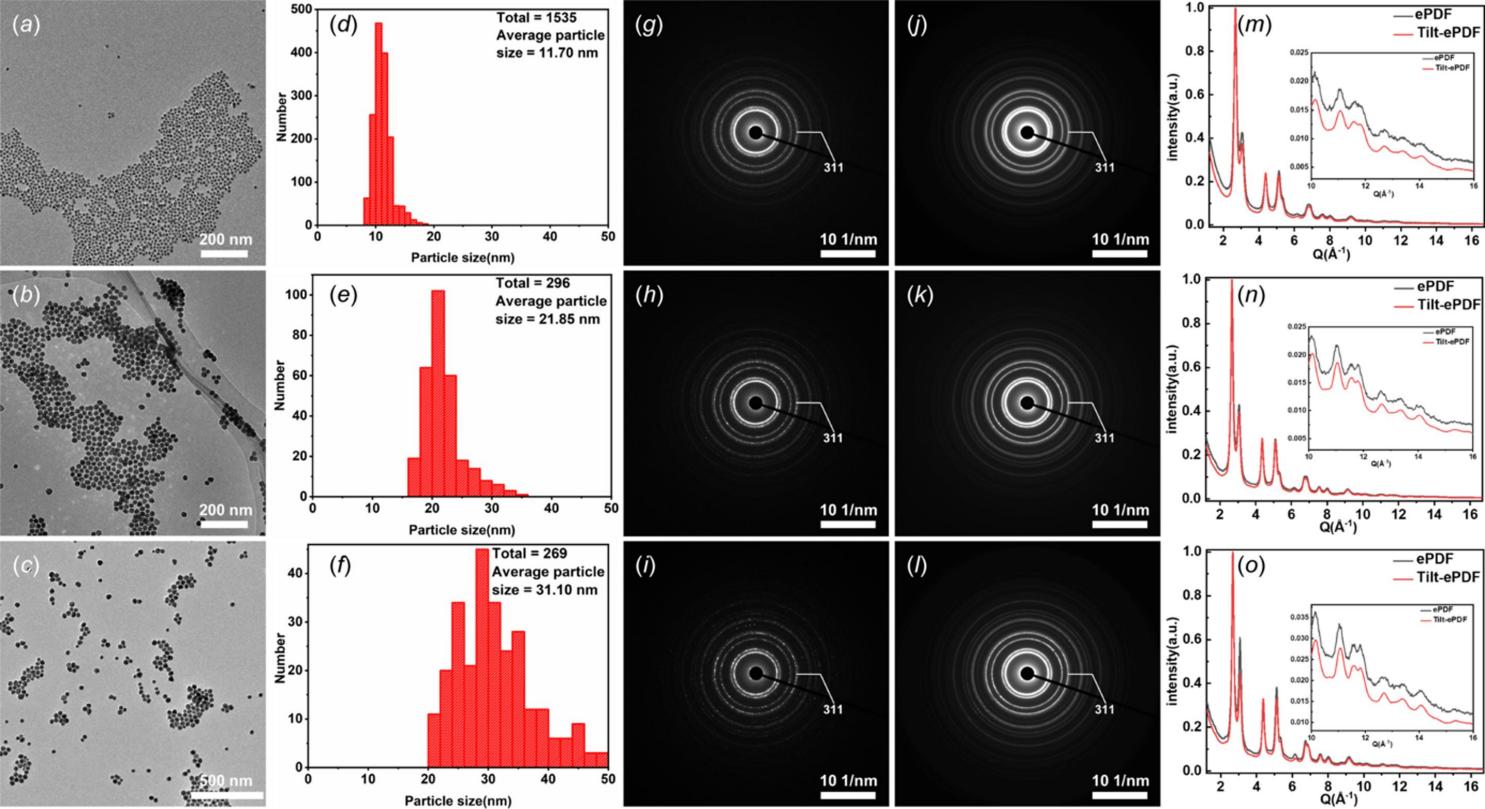
TEM images and ED data of three different gold nanoparticles. (*a*) TEM image of first AuNPs. (*b*) TEM image of second AuNPs. (*c*) TEM image of third AuNPs. (*d*) The average size of particles is ∼12 nm for the first AuNPs. (*e*) The average size of particles is ∼22 nm for the second AuNPs. (*f*) The average size of particles is ∼31 nm for third AuNPs. (*g*)–(*i*) Corresponding single-ePDF pattern of three kinds of AuNPs with a camera length of 250 mm. (*j*)–(*l*) Corresponding tilt-ePDF pattern of three kinds of AuNPs with a camera length of 250 mm. (*m*)–(*o*) 1D normalization intensity profile of three kinds of AuNPs from a single-ePDF and tilt-ePDF.

TEM images, as shown in Figs. 2 ▸(*a*)–2 ▸(*c*), display three different sizes of AuNPs. All AuNPs in these three samples are uniformly distributed on the carbon film, with most of them being spherical in morphology and there are crystalline domains in particles (Fig. S2), which is ideal for collecting ePDF data. We measured the particle size distribution using *Image-ProPlus 6.0* (https://mediacy.com/image-pro/). From the histogram of the AuNPs, the distribution range of first AuNPs is 8–16 nm with an average size of 12 nm [Fig. 2 ▸(*d*)]. The distribution range of second AuNPs is 16–30 nm, and most particles are distributed in the size range 18–24 nm [Fig. 2 ▸(*e*)]. The distribution range for the third AuNPs is 20–50 nm, which is less uniform than the previous two generations [Fig. 2 ▸(*f*)]. This is because the larger the AuNPs that are synthesized, the more difficult it is to control the morphology and size of the samples. The estimated average size of second and third AuNPs is 22 and 31 nm, respectively.

Diffraction patterns of single-ePDFs of AuNPs are illustrated in Figs. 2 ▸(*g*)–2 ▸(*i*) and tilt-ePDFs in Figs. 2 ▸(*j*)–2 ▸(*l*). As previously mentioned, the larger the nanoparticles, the smaller the number of particles that could be included in a fixed selected-area capture. Therefore, for a single SAED frame, with the increase of particle size, the discontinuousness of diffraction rings become obvious. Such a phenomenon can be clearly observed in Figs. 2 ▸(*g*)–2 ▸(*i*), that the diffraction rings were almost continuous with a moderate variation in intensity distribution [Fig. 2 ▸(*g*)] when the particle size is small, and the variation became more significant but maintains its ring shape [Fig. 2 ▸(*h*)] with the increase of particle size. The consistency of diffraction rings would be broken and the intensity distribution would be no longer uniform [Fig. 2 ▸(*i*)] as the particle size increases further. It is obvious that some diffraction rings are composed of a number of independent diffraction points.

As an advantageous data collection method, diffraction patterns of tilt-ePDFs in Figs. 2 ▸(*j*)–2 ▸(*l*) exhibit an obvious improvement of data quality. At small particle size, the diffraction rings showed a better consistency of intensity distribution [see Fig. 2 ▸(*j*)] compared with Fig. 2 ▸(*g*). And the diffraction rings were continuous as the size increased. For the largest particle size, the tilt-ePDF data not only kept the continuity of diffraction rings, but also showed an even intensity distribution. As can be seen from the 1D profiles [Figs. 2 ▸(*m*)–2 ▸(*o*)], the tilt-ePDF data have a better statistics and signal-to-noise ratio at high *Q* values compared with conventional ones.

**Figure 3:**
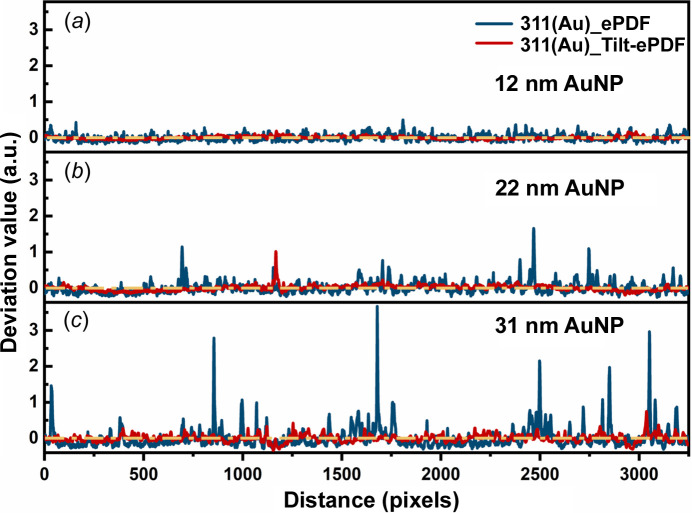
Intensity distribution of the 311 diffraction ring with different size AuNPs. Intensity distribution profiles of (*a*) first AuNPs, (*b*) second AuNPs and (*c*) third AuNPs from an SAED pattern and a tilt series of SAED patterns.

We chose the diffraction ring 311 as an example to measure the intensity distribution along the ring and calculate the normalized deviation values, as shown in Fig. 3 ▸. Ideally, the intensity distribution profile of a diffraction ring should be a straight line (yellow lines in Fig. 3 ▸). The diffraction ring of the first AuNPs [Fig. 3 ▸(*a*)] is close to the ideal one. However, as the size increases, some obvious peaks appear in the profiles of 311 diffraction rings from single SAED patterns, which come from individual particles with a large size [Figs. 3 ▸(*b*) and 3 ▸(*c*)]. In contrast, intensity distributions of 311 diffraction rings from tilt-ePDF are more uniform. These results show the advantages of tilt-ePDF compared with conventional ePDF.

**Figure 4:**
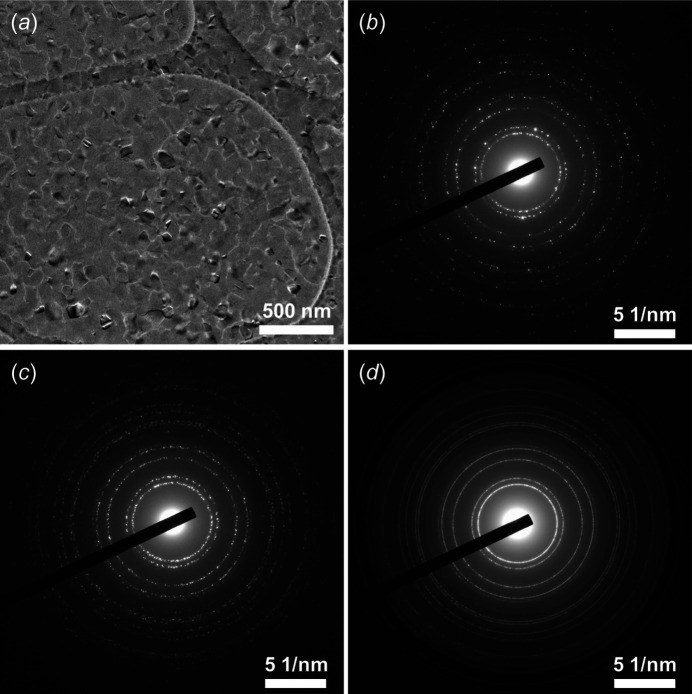
TEM images of the ED data of polycrystalline Al. (*a*) TEM image, (*b*) single-ED pattern, (*c*) PED pattern and (*d*) the merging of a tilt-series of ED patterns of polycrystalline Al with a camera length of 500 mm.

The polycrystalline Al and its corresponding diffraction data are shown in Fig. 4 ▸. The distribution of particle size is not optimal [see Fig. 4 ▸(*a*)], as a large number of big Al nanoparticles (≥100 nm) with polyhedral morphologies could be spotted, as a result of which the corresponding diffraction rings in ePDF data consist of many isolated spots [Figs. 4 ▸(*b*) and S1], the quality of diffraction patterns was observed as low for ePDF analysis. Compared with those in a single diffraction pattern and precession electron diffraction, the diffraction rings in tilt-ePDF are obviously more continuous [Figs. 4 ▸(*d*), S5 and S6].

### 3.2. The error range of the ePDF results

Before processing diffraction data of three AuNPs samples and polycrystalline Al film, it is necessary to perform a stability test on the ePDF data in order to reduce the instability factor and specify the error of the ePDF results. For stability testing, sputtered AuNPs were selected and prepared using an Ion Sputter SBC-12. The deposition process was completed once the entire carbon film was fully covered by the AuNPs, with a deposition time of approximately 20 s.

The deposited AuNPs on carbon film could be seen in Fig. S7, and most nanoparticles in the sample were uniformly distributed. Eight different areas on the carbon film were selected to collect the corresponding polycrystalline diffraction rings. The exposure time of each SAED frame is 10 s. The electron dose rate is kept at 1.900 e Å^−2^ s^−^^1^. The SAED patterns collected from eight areas show high consistency (Fig. S8) and little difference could be identified among these data. These diffraction data were further processed and refined against the standard structure model of Au (ICSD no. 44362).

From Table S4 and Fig. S9, the ePDF stability test results show that the absolute deviation Δ*a* (%) is less than ±0.35% and the error range of Δ*R*_w_ (%) is less than ±1%. It can be seen that the ePDF data obtained by collecting the diffraction rings of nanoparticles from different regions have a considerably high consistency and low deviation, and the results are reproducible.

### 3.3. ePDF refinement analysis

After Fourier transform, ePDF curves corresponding to different samples were obtained (for details, see the supporting information). The two major factors that determine the accuracy of *G*(*r*) are the diffraction intensity and the *Q* range. The larger the range of *Q* values recorded, the more accurate the diffraction intensity is, resulting in a more accurate result. The *Q*_max_ of our electron diffraction data is about 16.50 Å^−1^ and the optimal low scattering angle *Q*_min_ is chosen to reduce noise input from the central spot.

The refined parameters include particle size, cell parameters, atomic isotropic parameters (ADPs) *U* and decay factor *Q*_damp_. Since the particles size is already known, the diameter of the Au samples could be fixed at 12 nm (first AuNPs), 22 nm (second AuNPs) and 31 nm (third AuNPs) during the refinement. Meanwhile, to reduce the affecting influences, all datasets were processed using a similar fitting *Q* range with approximately 2.3–16.0 Å^−1^.

**Figure 5:**
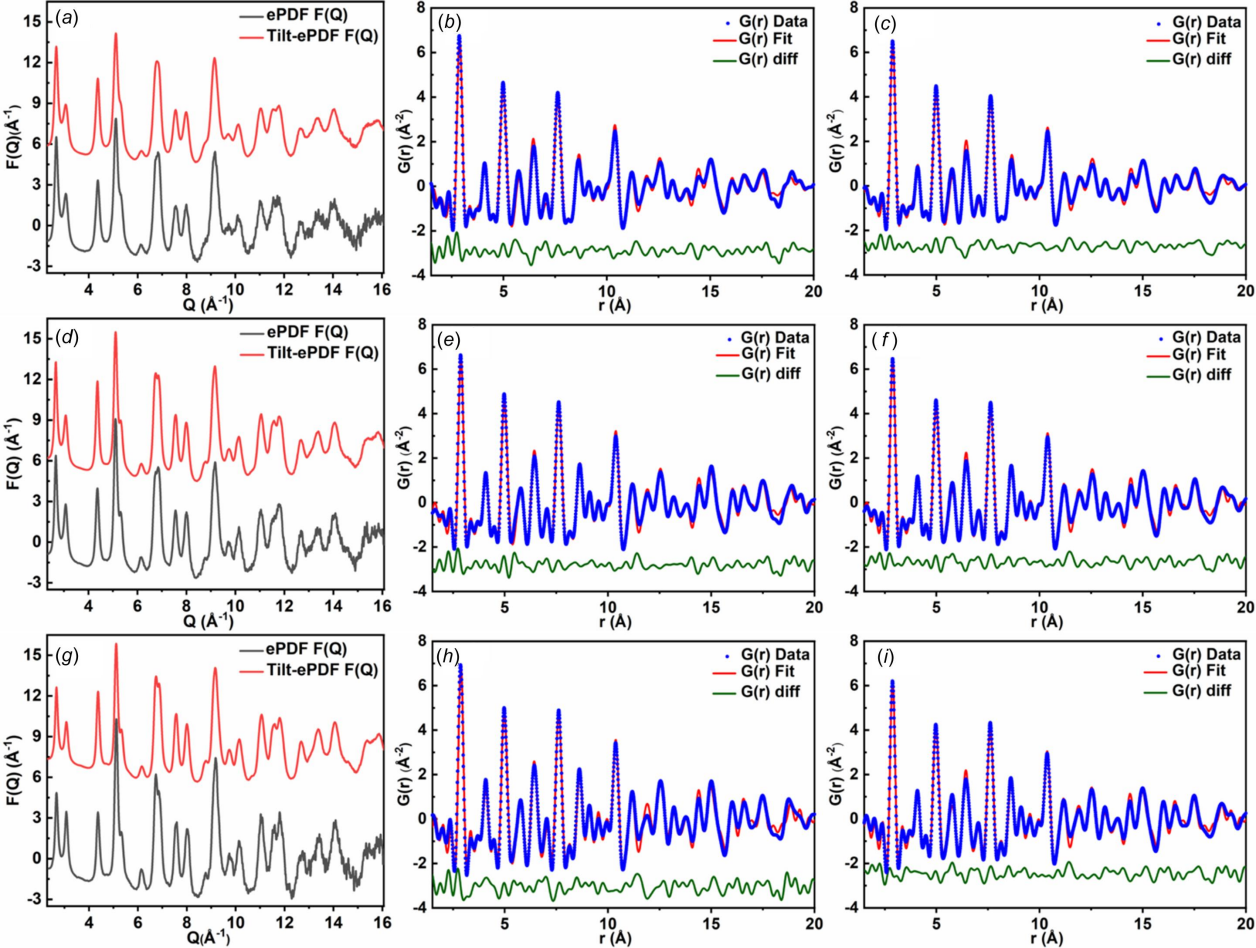
ePDF analysis of AuNPs. (*a*) The reduced structure function *F*(*Q*), *G*(*r*) of first AuNPs from (*b*) ePDF and (*c*) tilt-ePDF. (*d*) The reduced structure function *F*(*Q*), *G*(*r*) of second AuNPs from (*e*) ePDF and (*f*) tilt-ePDF. (*g*) The reduced structure function *F*(*Q*), *G*(*r*) of third AuNPs from (*h*) ePDF and (*i*) tilt-ePDF.

Compared with conventional ePDF, the reduce structure *F*(*Q*) profile [Fig. 5 ▸(*a*)] of the first AuNPs from tilt-ePDF is smoother and has a better signal-to-noise ratio in the high *Q* range (10–16 Å^−1^). Figs. 5 ▸(*b*) and 5 ▸(*c*) show the PDF fit analysis of the first AuNPs, and the refinement parameters are summarized in Tables S5 and S8. The cell parameters’ offset of single-ePDF and tilt-ePDF are −0.039 and −0.027%, respectively. These offsets are within the acceptable error range. A lower residual factor *R*_w_ (15.03%) was obtained from tilt-ePDF data, compared with 17.63% from single-ePDF. *Q*_Au_ and *Q*_damp_ are similar and consistent with previous reports (Abekyoon *et al.*, 2012 ▸; Schleder *et al.*, 2019 ▸), which indicates that the data are not over-fitted. The *F*(*Q*) of the second AuNPs is shown in Fig. 5 ▸(*d*). We can see that in the high *Q* range (10–16 Å^−1^), after the diffraction obtained by specimen tilting and data merging, the peaks displayed by *F*(*Q*) can be completely separated from the noise.

The corresponding refinement results of the second AuNPs could be found in Tables S6 and S9 and the ePDF fit analysis are showed in Figs. 5 ▸(*e*) and 5 ▸(*f*). The *Q*-range was set to be same as that of the first AuNPs, and the tilt-ePDF data exhibited a better result, with a lower *R*_w_ values of 14.60%, compared with those from single-ePDF (15.83%). In addition, compared with the first AuNPs, the *U*_Au_ of the second AuNPs decreases by about 20%, which indicates that the atoms gradually change from disordered to ordered during the growth of AuNPs. The data for the third AuNPs also showed that the *F*(*Q*) from tilt-ePDF is better than that of the conventional technique in the high *Q* range. In the ePDF refinement [Figs. 5 ▸(*h*) and 5 ▸(*i*); Tables S7 and S10], the offsets of unit-cell parameter *a* are close for ePDF and tilt-ePDF, both of which are within the error range. Moreover, it seems that tilt-ePDF data are less affected by multiple scattering effects (Fig. S10). The lower *U*_Au_, which is again reduced by about 20%, also indicates that the structure is more ordered than the previous two generations of AuNPs. In addition, the *R*_w_ value was reduced from 19.62 to 16.98%.

Given that the ePDF refinement results for the three different sizes of nanoparticles lead to nearly a 3%_w_ reduction in *R*_w_, it had proven that the method of tilt-ePDF has the capability to improve data quality and produce more accurate structure refinement results. In addition, we also calculate the refined PDF data (Tables S11–S13) by merging different numbers of diffraction patterns from a tilt-series. Apparently, compared with a single ED pattern, the merging of multiple diffraction patterns reduced the residual factors. However, there is no universal rule for how many patterns should be used for merging, which might vary depending on the samples.

**Figure 6:**
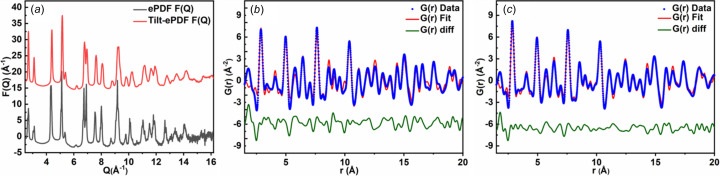
ePDF analysis of polycrystalline Al film. (*a*) Reduced structure function *F*(*Q*). *G*(*r*) from (*b*) ePDF and (*c*) tilt-ePDF.

Similar results were also obtained from ePDF analysis of polycrystalline Al film. In the range of *Q* values (10–16 Å^−1^), the *F*(*Q*) profile from tilt-ePDF is smoother than that of ePDF [Fig. 6 ▸(*a*)]. The refinement of *G*(*r*) profiles was performed against a standard structural model. Compared with conventional ePDF data, tilt-ePDF data show a better fitting with the structural model, as revealed from lower *R*_w_ factors and lower unit-cell parameter deviations (Tables S14–S16). To study the effects of texture and crystal thickness on agreement factors in the ePDF analysis, the ePDF data of three Al film samples with different thicknesses (∼20, 60 and 150 nm) are analyzed. As the thickness of the Al film increases, the *R*_w_ value also increases. In particular, the *R*_w_ value of 150 nm-thick Al film increases to 52.76%. The results show that the thickness of the Al film is the main factor affecting the agreement parameter (Table S17). Moreover, tilt-ePDF data also show advantages for the further analysis of bond lengths and coordination numbers (Table S18).

## 4. Conclusions

To overcome the size limitation for nanoparticles in the implementation of ePDF, a new method, tilt-ePDF, was proposed by combining ED with specimen tilting. A tilt-series of ED patterns was collected from multiple nanocrystals with continuous tilting of the specimen. As a result, diffraction rings became more consecutive compared with those in single ED and PED patterns, and the signal-to-noise ratio was also improved, especially in the high-scattering-angle range. A better fitting with the structural model was obtained during the following refinement. These results confirm that the tilt-ePDF method facilitates the application of the ePDF method to large-sized nanoparticles, thus broadening the scope of this technique. With the rapid development of ePDF, the new method proposed here might provide an additional way to obtain quantitative structural information from nanoparticles.

## 5. Related literature

The following references are cited in the supporting information: Schneider *et al.* (2012 ▸); Proffen & Neder (1999 ▸); Page *et al.* (2011 ▸); Juhás *et al.* (2015 ▸).

## Supplementary Material

Supporting information file. DOI: 10.1107/S2052252524001064/of5003sup1.pdf
