# Supplementary material for: Structural analysis of nanocrystals by pair distribution function combining electron diffraction with crystal tilting
Source: IUCrJ. 2024 Feb 16;11(Pt 2):202–9. doi: 10.1107/S2052252524001064 (PMC10916296; doi:10.1107/S2052252524001064)
Supplement: Supplementary file 1 [file m-11-00202-sup1.pdf]

IUCrJ

Volume 11 (2024)

Supporting information for article:

Structural analysis of nanocrystals by pair distribution function combining electron diffraction with crystal tilting

Linshuo Guo, Shitao Wu, Zhengyang Zhou and Yanhang Ma

Supporting information

Structural Analysis of Nanocrystals by Pair Distribution Function Combining Electron Diffraction with Crystal Tilting

Linshuo Guo[a], Shitao Wu[a*], Zhengyang Zhou[b*] and Yanhang Ma[a*]

[a] School of Physical Science and Technology & Shanghai Key Laboratory of High-resolution Electron Microscopy, ShanghaiTech University, Shanghai, 201210, People's Republic of China
[b] State Key Laboratory of High Performance Ceramics and Superfine Microstructure, Shanghai Institute of Ceramics, Chinese Academy of Sciences, Shanghai, 200050 People's Republic of China

Correspondence email: wusht@shanghaitech.edu.cn; zhouzhengyang@mail.sic.ac.cn; mayh2@shanghaitech.edu.cn

S1. Detalis of materials and methods

S1.1. Evaporated Aluminium

Table S1 The reflection indices and the corresponding scattering vector *Q* of standard sample polycrystalline Al obtained from the Ted Pella Inc. The lattice parameter *a* is 4.0494 Å.

hkl	*Q* (Å^{-1})	intensity
111	2.69	100
200	3.10	47
220	4.39	22
311	5.15	24
222	5.38	7

S1.2. Data collection of single-ePDF and tilt-ePDF

Table S2 Tilt-ePDF data collection parameters.

sample	rotation rate (°/frame)	rotation angle (°)	dose rate (e Å^{2-} s^{-1})
1st AuNPs	0.3663	65.934	1.7405
2st AuNPs	0.3617	65.106	1.7459
3st AuNPs	0.3465	62.370	1.8980
polycrystalline Al	0.3273	58.910	1.5947

Table S3 Details of diffraction patterns in single-pattern ePDF and tilt-ePDF.

-	1st AuNPs	2nd AuNPs	3rd AuNPs	polycrystalline Al
Single-pattern size	4093*4094	4093*4094	4094*4092	4096*4096
Single-pattern center	(2041,2024)	(2037,2032)	(2036,2038)	(2042,2039)
Tilt-pattern size	4093*4094	4093*4094	4094*4092	4093*4095
Tilt-pattern center	(2047,2031)	(2037,2032)	(2035,2038)	(2046,2041)

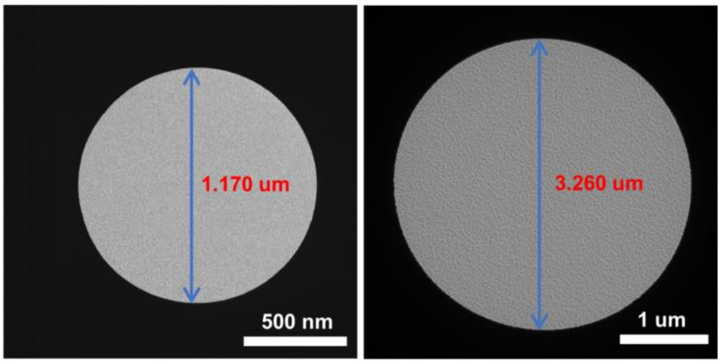

Figure S1 (*a*) TEM image of illumination area using the largest SAED aperture in JEOL JEM-F200. (*b*) TEM image of illumination area using the largest SAED aperture in JEOL JEM-2100Plus.

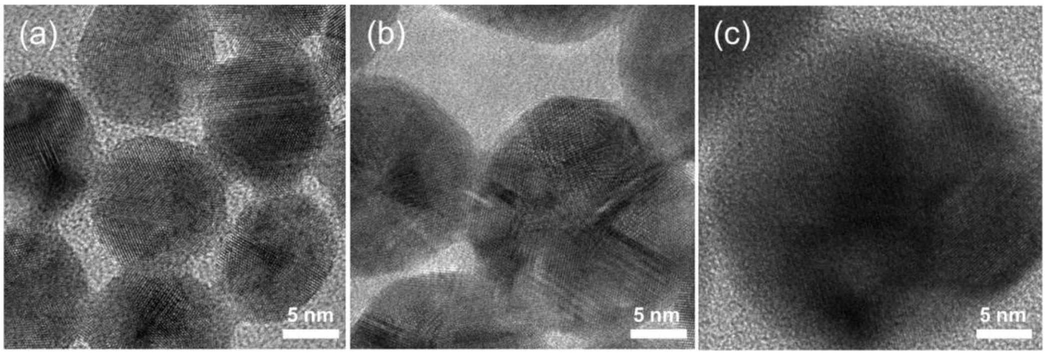

Figure S2 HRTEM image of (*a*) 1st AuNPs, (*b*)2nd AuNPs and (*c*) 3rd AuNPs.

S1.3. The background subtraction

Background fitting of the raw data in natural logarithmic transformation (NLT) was chosen to avoid overfitting at high Q values and losing the corresponding diffraction data. The background is subtracted using the following procedures.

1. The 2D diffraction data was imported into the *ImageJ* software (Caroline A Schneider *et al.,* 2012), and the center 150 pixels were masked. 1D intensity profile (Figure S3*a*) was obtained from the 2D diffraction ring using the program *ImageJ-Radial profile*.

2. The 1D intensity profile was transformed using NLT function (black line in Figure S3b) and the corresponding background (red line in Figure S3*b*) was obtained using program "*peaks and baseline*" in the software *Origin*.

3. An inverse logarithmic transformation (exponential function) was applied to red line in Figure S3b to obtain the red line in Figure S3*c*.

4. Figure S3*d* is the data after removing background. (Figure S3*d* = black line in Figure S3*c* -red line in Figure S3*c*.

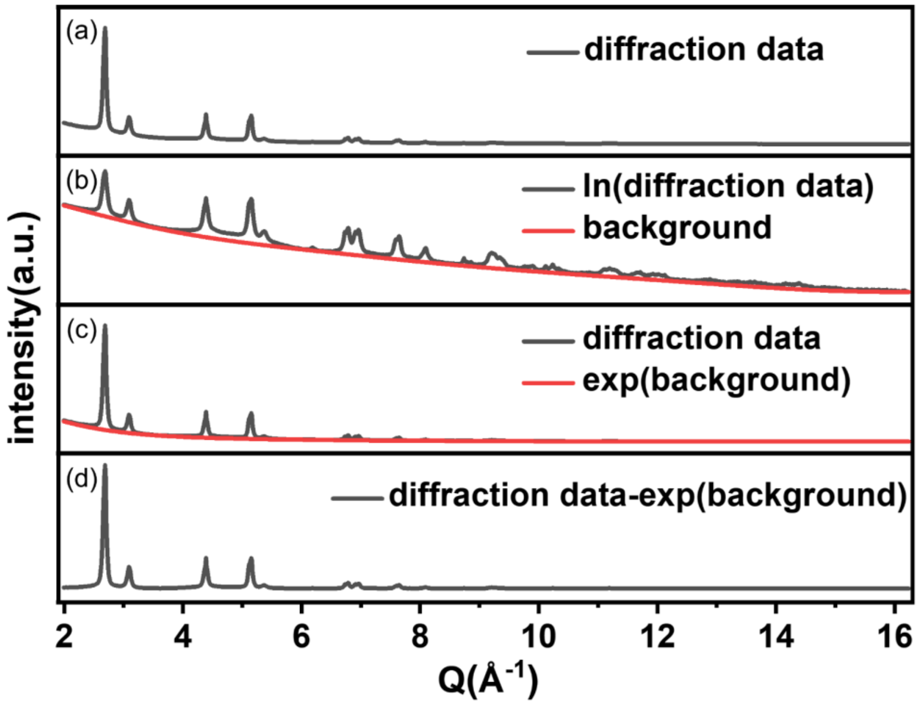

Figure S3 The procedure of removing the background from 1D scattering intensity profile of polycrystalline Al film. (*a*) The raw 1D intensity profile. (*b*) The raw 1D intensity profile after NLT (black) and background profile (red) calculated using "peaks and baseline" in the software Origin. (*c*) Black profile is same as Figure S3*a* and red profile is obtained from Figure S3*b*'s red lines through inverse NLT. (*d*) The data after removing background.

S1.4. The Principle of Pair Distribution Function

The ePDF could be calculated by Fourier transformation of the sample diffraction data. The fundaments of in data processing procedures are elucidated as follows.

1. One-dimensional integration of collected diffraction raw data with optimized processing and normalization. After subtracting the diffuse background, the intensity normalization transformation $I(Q)$ (Figure S4*a*) could be completed using Eq. (1).

$$I(Q)=\frac{\int_{Q_{min}}^{Q_{max}}\left\langle f_e^2(Q)\right\rangle dQ}{\int_{Q_{min}}^{Q_{max}}(I_{rawdata}-I_{background})dQ}[(I_{rawdata}-I_{background})] \qquad (1)$$

where $Q = 4\pi sin(\theta)/\lambda$ is the scattering vector, $f_e(Q)$ is the atomic scattering factor. Because noise has an effect on both low Q and high Q, it is worth-considering to select a suitable Q range. $I_{rawdata}$ is experimental scattering intensity.

2. Calculation of *S*(*Q*) and *F*(*Q*) were using the obtained normalized diffraction *I*(*Q*). $S(Q)$ (Figure S4*b*) is the total structure factor and $F(Q)$ (Figure S4*c*) is the reduced structure function, which can be calculated from $S(Q)$. The calculation method could be seen in Eq. (2) and Eq. (3).

$$S(Q)=\frac{I(Q)-\left\langle f_e^2(Q)\right\rangle}{\langle f_e(Q)\rangle^2}+1 \qquad (2)$$

$$F(Q)=\left[\frac{I(Q)-\left\langle f_e^2(Q)\right\rangle}{\langle f_e(Q)\rangle^2}\right]Q\leftrightarrow F(Q)=Q[S(Q)-1] \qquad (3)$$

3. Calculations of reduced PDF *G*(*r*). The ePDF $G(r)$ (Figure S4*d*) represent the probability of finding two atoms with the distance of r. The PDF processing and related results are discussed in a *DISCUS_Suite* software (Proffen & Neder, 1999; Page *et al.,* 2011). $G(r)$ could be calculated as follows,

$$G(r)=4\pi r[\rho(r)-\rho_a]=\frac{2}{\pi}\int_{Q_{min}}^{Q_{max}}F(Q)\sin(Q\cdot r)\,dQ \qquad (4)$$

where $\rho(r)$ is the atomic pair density, ρ_a is the average atomic number density, and r is the radial distance.

4. The refinement of ePDF. In order to compare ePDF data with different collection methods and different particle sizes, we refined the reduced PDF $G(r)$ of each sample to estimate the quality of the data. The standard models are simulated with a standard unit cell by assuming spherical AuNPs with different sizes, while for polycrystalline Al specimen, the standard model is considered as bulk. The structure refinement of the ePDF data was processed through a least square fitting approach using the *DiffPy-CMI* program (Juhás *et al.,* 2015).

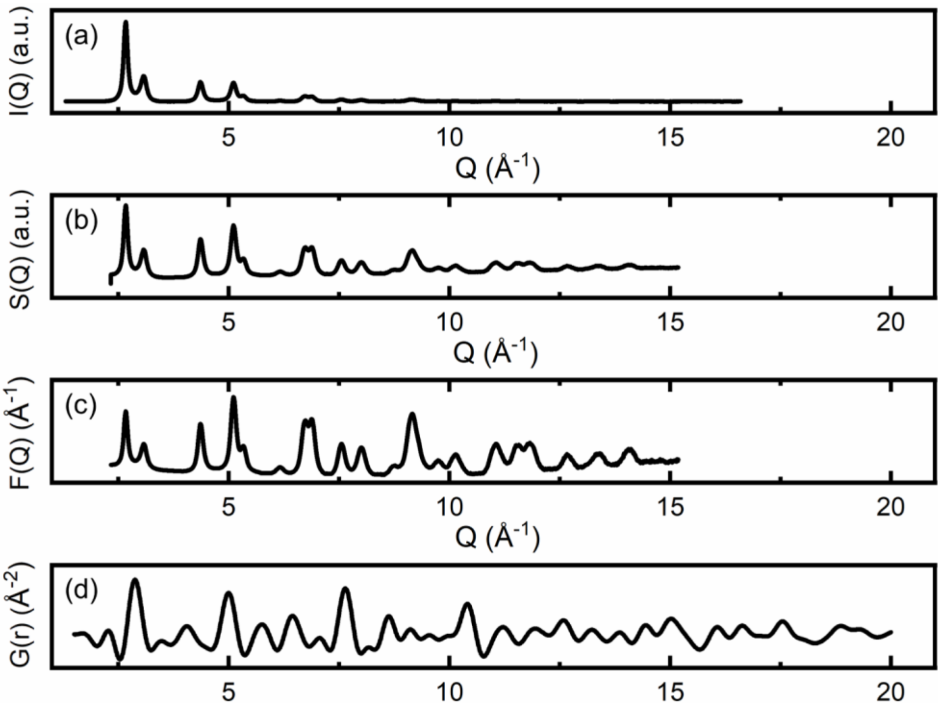

Figure S4 Procedure of ePDF analysis from ED data. (*a*) 1D integrated diffraction data after diffuse background subtraction. (*b*) The total structure factor *S*(*Q*) of AuNPs from ED pattern. (*c*) The reduced structure function *F*(*Q*). (*d*) The reduced pair distribution function *G*(*r*).

S2. Details of results

S2.1. Electron diffraction

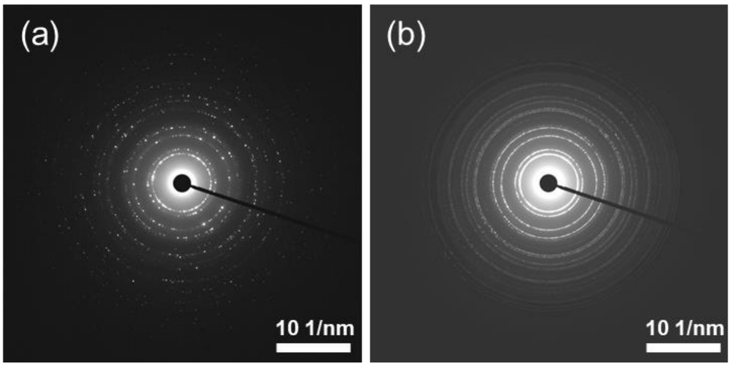

Figure S5 (a) A single ED pattern and (b) merging a tilt-series of ED patterns of polycrystalline Al (recorded using F200 instrument and camera length 250 mm).

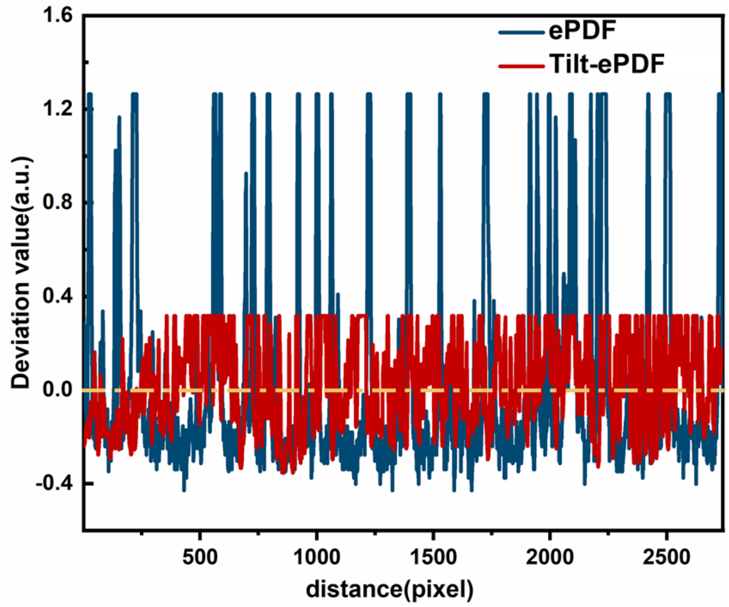

Figure S6 The intensity distribution of 220 diffraction ring of polycrystalline Al film from a single ED pattern and a tilt-series of ED patterns respectively.

S2.2. The error range of ePDF results

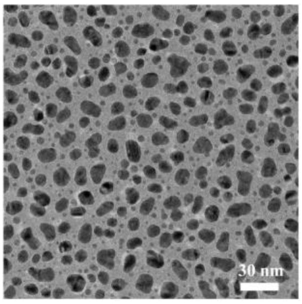

Figure S7 TEM image of the Au nanoparticle sample obtained from ion sputtering.

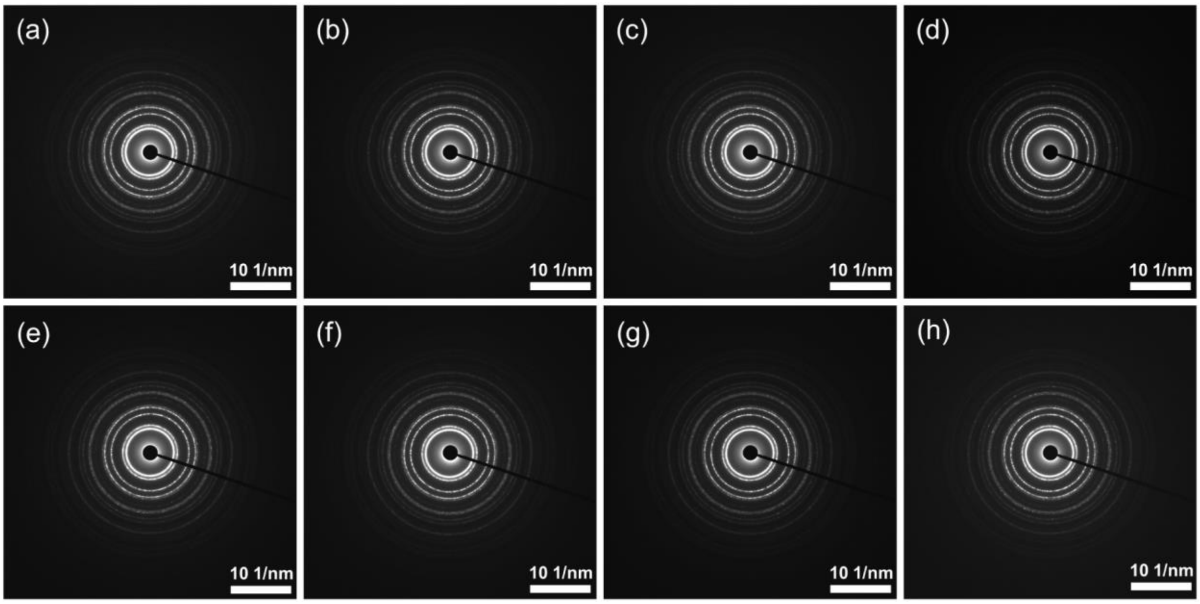

Figure S8 The eight SAED patterns from different regions of AuNPs obtained from ion sputtering.

Table S4 The ePDF refinement results using eight ED patterns in Figure S8.

frame	R_w (%)	a (Å)	ΔR_w (%)	Δa (Å)	Δa (Å)%
1	21.0	4.0812	-0.7951	-0.0016	-0.0392
2	21.3	4.0693	-0.4298	0.0103	0.2525
3	22.0	4.0710	0.2168	0.0086	0.2108
4	21.4	4.0706	-0.3368	0.0090	0.2206
5	22.4	4.0657	0.6738	0.0139	0.3407
6	22.2	4.0680	0.4770	0.0116	0.2843
7	21.0	4.0781	-0.7293	0.0015	0.0368
8	22.7	4.0789	0.9235	0.0007	0.0172

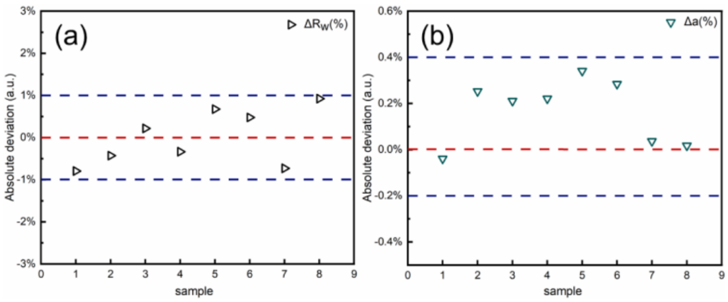

Figure S9 The plots of ΔR_w (%) and Δa (%) distribution in Table S4.

S2.3. Details of ePDF refinement analysis

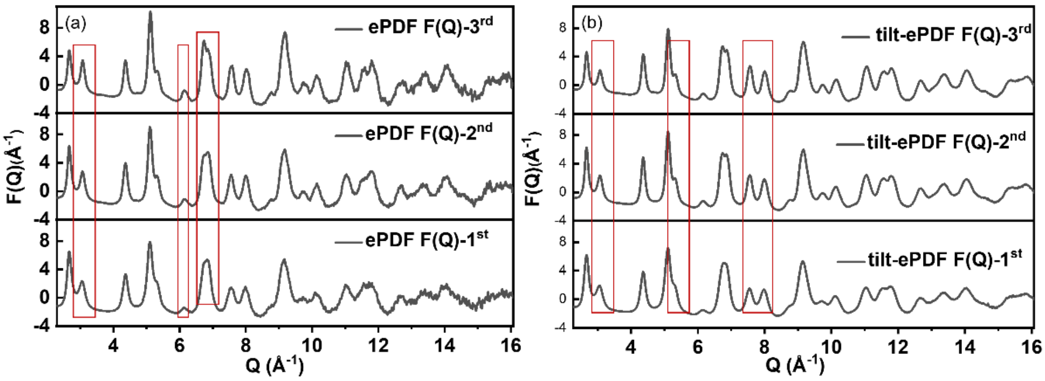

Figure S10 The reduced structure function *F(Q)* of 1st, 2rd, and 3nd AuNPs from (*a*) ePDF and (*b*) tilt-ePDF. As shown in Figure S10, we can see that F(Q) values change with the particle size, which can be attributed to multiple scattering effects. For example, intensity of the second peak in Figure S10a gradually increases relative to the first peak with the increase of particle size. Moreover, it seems that tilt-ePDF data are less affected by multiple scattering effects, as shown in Figure S10b.

Table S5 The refinement results of 1st Au NPs.

Au	Single-ePDF	Tilt-ePDF	Ref.a
a (Å)	4.0780	4.0785	4.0796
Δ*a* (Å)%	-0.039%	-0.027%	-
U_{Au} (Å^2)	0.04017	0.0394	-
Q_{damp} (Å^{-1})	0.1021	0.1003	-
R_w (%)	17.63	15.03	-
Q-range (Å^{-1})	2.297-16.306	2.281-16.076	-

Table S6 The refinement results of 2nd Au NPs.

Au	Single-ePDF	Tilt-ePDF	Ref.a
a (Å)	4.0724	4.0766	4.0796
Δ*a* (Å)%	-0.18%	-0.074%	-
U_{Au} (Å^2)	0.0325	0.0336	-
Q_{damp} (Å^{-1})	0.0954	0.0923	-
R_w (%)	15.83	14.60	-
Q-range (Å^{-1})	2.298-16.117	2.304-16.088	-

Table S7 The refinement results of 3rd Au NPs.

Au	Single-ePDF	Tilt-ePDF	Ref.a
a (Å)	4.0696	4.0672	4.0796
Δa (Å)%	-0.25%	-0.30%	-
U_{Au} (Å^2)	0.0266	0.0280	-
Q_{damp} (Å^{-1})	0.0961	0.0927	-
R_w (%)	19.62	16.98	-
Q-range (Å^{-1})	2.345-16.108	2.313-16.066	-

Table S8 The refinement results of 1st Au NPs using ePDF data collected with different methods.

Au	Single-ePDF	PED-ePDF	Tilt-ePDF	Ref.a
a (Å)	4.0776	4.0812	4.0807	4.0796
Δa (Å)%	-0.049%	0.039%	0.027%	-
U_{Au} (Å^2)	0.0438	0.0438	0.0540	-
Q_{damp} (Å^{-1})	0.0889	0.1008	0.1001	-
R_w (%)	18.30%	16.81%	17.62%	-
Q-range (Å^{-1})	2.223-11.543	2.248-11.629	2.246-11.607	-

Table S9 The refinement results of 2nd Au NPs using ePDF data collected with different methods.

Au	Single-ePDF	PED-ePDF	Tilt-ePDF	Ref.a
a (Å)	4.0835	4.0830	4.0779	4.0796
Δa (Å)%	0.096%	0.083%	-0.042%	-
U_{Au} (Å^2)	0.0351	0.0356	0.0345	-
Q_{damp} (Å^{-1})	0.0936	0.0983	0.0922	-
R_w (%)	16.96%	16.17%	15.91%	-
Q-range (Å^{-1})	2.281-11.623	2.288-11.645	2.272-11.647	-

Table S10 The refinement results of 3rd Au NPs using ePDF data collected with different methods.

Au	Single-ePDF	PED-ePDF	Tilt-ePDF	Ref.a
a (Å)	4.0842	4.0871	4.0751	4.0796
Δa (Å)%	0.113%	0.184%	-0.110%	-
U_{Au} (Å^2)	0.0280	0.0283	0.0236	-
Q_{damp} (Å^{-1})	0.0835	0.0869	0.0913	-
R_w (%)	22.02%	21.34%	22.00%	-
Q-range (Å^{-1})	2.301-11.597	2.317-11.570	2.161-11.620	-

Table S11 The refinement results of 1st Au NPs by merging different numbers of ED frames from a tilt-series.

Number of frames	R_w (%)	Lattice parameter (Å)
1	17.63	4.0780
30	16.15	4.0776
60	16.14	4.0775
90	16.38	4.0649
120	15.27	4.0775
150	14.87	4.0774
180	15.03	4.0785

Table S12 The refinement results of 2nd Au NPs by merging different numbers of ED frames from a tilt-series.

Merge frame	R_w (%)	Lattice parameter (Å)
1	15.83	4.0724
30	15.74	4.0723
60	15.50	4.0727
90	15.87	4.0730
120	15.13	4.0734
150	14.52	4.0739
180	14.60	4.0766

Table S13 The refinement results of 3rd Au NPs by merging different numbers of ED frames from a tilt-series.

Merge frame	R_w (%)	Lattice parameter (Å)
1	19.62	4.0696
30	17.39	4.0699
60	16.90	4.0816
90	16.63	4.0685
120	16.03	4.0673
150	17.10	4.0680
180	16.98	4.0672

Table S14 The refinement results of polycrystalline Al.

Al	Single-ePDF	Tilt-ePDF	Ref.a
a (Å)	4.0766	4.0422	4.0494
Δa (Å)%	0.67%	-0.18%	-
U_{Au} (Å^2)	0.0291	0.0370	-
Q_{damp} (Å^{-1})	0.0713	0.0661	-
R_w (%)	33.58	22.98	-
Q-range (Å^{-1})	2.429-16.331	2.445-16.176	-

Table S15 The refinement results of Al film by merging different numbers of ED frames.

Merge frame	R_w (%)	Lattice parameter (Å)
1	33.58	4.0766
30	24.65	4.0427
60	22.80	4.0457
90	23.26	4.0485
120	24.60	4.0502
150	23.47	4.0401
180	22.98	4.0422

Table S16 The refinement results of Al film using ePDF data collected with different methods.

Al film	Single-ePDF	PED-ePDF	Tilt-ePDF	Ref.a
a (Å)	4.0529	4.0392	4.0512	4.0494
Δa (Å)%	0.086%	-0.252%	0.045%	-
U_{Au} (Å^2)	0.0235	0.0267	0.0258	-
Q_{damp} (Å^{-1})	0.0692	0.0667	0.0686	-
R_w (%)	30.52	26.93	22.91	-
Q-range (Å^{-1})	2.412-11.763	2.434-11.766	2.365-11.695	-

Table S17 The ePDF refinement results of Al film with different thicknesses.

Al film	Tilt-ePDF_20 nm	Tilt-ePDF _60 nm	Tilt-ePDF_150 nm	Ref.a
a (Å)	4.0506	4.0510	4.0481	4.0494
Δa (Å)%	0.030%	0.040%	-0.032%	-
U_{Au} (Å^2)	0.0549	0.0421	0.0185	-
Q_{damp} (Å^{-1})	0.0682	0.0719	0.0777	-
R_w (%)	14.16	16.08	52.76	-
Q-range (Å^{-1})	2.346-11.682	2.246-11.635	2.130-11.695	-

Table S18 Calculated bond lengths and coordination numbers (CNs) of polycrystalline Al, and comparison with data from the standard model.

Coordination sphere	Single-ePDF		Tilt-ePDF		Ref.ePDF	
	Bond length(Å)	CNs	Bond length(Å)	CNs	Bond length(Å)	CNs
1 (2.64-3.13) / (2.62-3.11)	2.89	11.53	2.86	11.48	2.8633	12
2 (3.59-4.36) / (3.58-4.36)	4.09	7.14	4.06	6.95	4.0494	6
3 (4.66-5.34) / (4.58-5.34)	4.99	22.83	4.95	22.65	4.9595	24
4 (5.42-6.10) / (5.34-6.04)	5.78	12.48	5.73	11.75	5.72672	12
5 (6.10-6.79) / (6.04-6.75)	6.45	24.90	6.39	24.53	6.40266	24
6 (6.79-7.28) / (6.75-7.19)	7.07	9.59	7.00	8.51	7.01337	8

References

Caroline A Schneider., Wayne S Rasband., Kevin W Eliceiri. (2012). *Nature Methods*. **9**, 671–675.

Origin 2020. https://www.originlab.com/.

Th. Proffen., R. B. Neder. (1999). *J. Appl. Cryst.* **32**, 838-839.

Katharine Page., Taylor C. Hood., Thomas Proffen., Reinhard B. Nederb. (2011). *J. Appl. Cryst.* **44**, 327-336.

P. Juhás., C. Farrow., X. Yang., K. Knox., S. Billinge. (2015). *Acta Cryst. A***71**, 562-568.
